# Supplementary material for: Traumatic Fracture Treatment: Calcium Phosphate Bone Substitute Case–Control Study in Humerus, Radius, Tibia Fractures—Assessing Efficacy and Recovery Outcomes
Source: Biomedicines. 2023 Oct 22;11(10):2862. doi: 10.3390/biomedicines11102862 (PMC10604612; doi:10.3390/biomedicines11102862)
Supplement: Supplementary file 1 [file biomedicines-11-02862-s001.zip › biomedicines-2539492-supplementary.pdf]

Supplementary Data:

Table S1. Radiological criteria for bone healing, modified after Bohndorf et al. [28], Freyschmidt et al. [29], and Islam et al. [30].

Criterion:	Classification:	Radiological criteria:
Edge of fracture	1	No visible edges
	2	Dimly visible edges
	3	Blurred edge
	4	Partly sharp-and partly blurred-edged
	5	Sharp-edged
Fracture gap	1	No fracture gap: Bridging is completed
	2	Condensation
	3	Partly blurred and partly compressed
	4	Blurred
	5	Reduction in density and bone gap looks more impressive
	6	Visible gap
Articular surface	1	No anomalies
	2	Intraarticular fracture gap
	3	Intraarticular stage
	4	Intraarticular stage and intraarticular fracture gap
Osteosynthesis	1	Explanation of osteosynthesis because of bone healing
	2	All fracture fragments are fixed, osteosynthesis screws are extraarticular, osteosynthesis plate is fitted tightly on the corticalis, and no material fracture
	3	All fracture fragments are fixed, osteosynthesis screws are extraarticular, osteosynthesis plate is fitted tightly on the corticalis, no material fracture, X-ray image while wearing gypsum
	4	Osteosynthesis screws are extraarticular, osteosynthesis plate is fitted tightly on the corticalis, no material fracture, and some fracture fragments are not fixed
	5	Osteosynthesis screws are extraarticular, osteosynthesis plate is fitted tightly on the corticalis, no material fracture, some fracture fragments are not fixed, X-ray image while wearing gypsum
	6	Pathological damages at the osteosynthesis, e.g.,:

		- Plate is not fitting tightly to the corticalis<br>- Screws are intraarticular<br>- other pathology
Bone structure	1	Normal bone substance
	2	Local condensation
	3	Reduction of radiopaque at fracture tilt
	4	Resorption of the fracture callus
